# Development and preclinical evaluation of cixutumumab drug conjugates in a model of insulin growth factor receptor I (IGF-1R) positive cancer

**DOI:** 10.1038/s41598-020-75279-z

**Published:** 2020-10-29

**Authors:** Viswas Raja Solomon, Elahe Alizadeh, Wendy Bernhard, Amal Makhlouf, Siddesh V. Hartimath, Wayne Hill, Ayman El-Sayed, Kris Barreto, Clarence Ronald Geyer, Humphrey Fonge

**Affiliations:** 1 Department of Medical Imaging, RUH Saskatoon, University of Saskatchewan, College of Medicine, 103 Hospital Dr., Saskatoon, SK S7N 0W8 Canada; 2 Department of Pathology and Laboratory Medicine, University of Saskatchewan, College of Medicine, Saskatoon, SK Canada; 3 Department of Pharmaceutics and Industrial Pharmacy, Faculty of Pharmacy, Cairo University, Kasr El-Aini, Cairo, 12411 Egypt; 4 Department of Medical Imaging, Royal University Hospital Saskatoon, Saskatoon, SK Canada

**Keywords:** Receptor pharmacology, Drug delivery

## Abstract

Overexpression of insulin growth factor receptor type 1 (IGF-1R) is observed in many cancers. Antibody drug conjugates (ADCs) with PEGylated maytansine (PEG_6_-DM1) show promise in vitro. We developed PEG_6_-DM1 ADCs with low and high drug to antibody ratios (DAR) using an anti-IGF-1R antibody cixutumumab (IMC-A12). Conjugates with low (cixutumumab-PEG_6_-DM1-Low) and high (cixutumumab-PEG_6_-DM1-High) DAR as 3.4 and 7.2, respectively, were generated. QC was performed by UV spectrophotometry, HPLC, bioanalyzer, and biolayer-interferometry. We compared the in vitro binding and internalization rates of the ADCs in IGF-1R-positive MCF-7/Her18 cells. We radiolabeled the ADCs with ^111^In and used microSPECT/CT imaging and ex vivo biodistribution to understand their in vivo behavior in MCF-7/Her18 xenograft mice. The therapeutic potential of the ADC was studied in vitro and in mouse xenograft. Internalization rates of all ADCs was high and increased over 48 h and EC_50_ was in the low nanomolar range. MicroSPECT/CT imaging and ex vivo biodistribution showed significantly lower tumor uptake of ^111^In-cixutumumab-PEG_6_-DM1-High compared to ^111^In-cixutumumab-PEG_6_-DM1-Low and ^111^In-cixutumumab. Cixutumumab-PEG_6_-DM1-Low significantly prolonged the survival of mice bearing MCF-7/Her18 xenograft compared with cixutumumab, cixutumumab-PEG_6_-DM1-High, or the PBS control group. Cixutumumab-PEG_6_-DM1-Low ADC was more effective. The study highlights the potential utility of cixutumumab-ADCs as theranostics against IGF-1R positive cancers.

## Introduction

Insulin growth factor receptor type 1 (IGF-1R) plays a key role in normal growth and development. Altered expression of IGF-1 pathway is implicated in the development and maintenance of malignant phenotypes in many primary cancers (such as lung, colorectal, pancreatic, breast, and colon cancers and glioblastomas) and their metastases, suggesting that imaging and therapeutic agents targeting IGF-1R receptor have a potential for diagnosis and therapy^1–4^. In addition to their role in these cancers, a cross-talk between IGF-1R and other growth factor receptors, notably epidermal growth factor receptors (HER family) has been implicated in the development of resistance to targeted therapies^4–7^. Cell-line data suggest that signaling through IGF-1R and downstream pathways are a mechanism of resistance to HER2 tyrosine kinase inhibitors (TKIs) and monoclonal antibodies (mAb), and that targeting both pathways leads to synergistic decreases in downstream signaling^7,8^. In the last decade many clinical trials using mAb and TKIs against IGF-1R were initiated, including ganitumab, figitumumab, and cixutumumab^9,10^. However, many of these agents failed to show any improvement in clinical outcomes. The reasons for the failed trials have been extensively reviewed^10^. Therefore, more effective therapeutic agents against this target are needed.

The efficacy of these antibodies can be improved by conjugation of multiple cytotoxic agents to the antibody. Anti-HER2 antibody trastuzumab conjugated to cytotoxic agent maytansine (DM1) has shown efficacy in preclinical models and in patients^11,12^. In trastuzumab-DMI (T-DM1; Kadcyla^®^), the antibody is conjugated to DM1 via a non-cleavable linker *N*-maleimidomethyl cyclohexane-1-carboxylate (MCC). However, overall response rate was with T-DM1 is a modest 35%^13–15^. Resistance to T-DM1 and other ADC is widespread^16,17^. Factors responsible for resistance include low antigen expression, receptor down-regulation, inefficient lysosomal degradation of T-DM1 and most notably the expression of drug efflux pump multidrug resistant gene (MDR1). Small molecules such as DM1 are substrates for MDR1 and readily pumped out of the cell via PgP^16–18^. Kovtun et al. and others showed that PEGylated DM1 (PEG-DM1) is not a MDR1 substrate^16–18^.

Cixutumumab (IMC-A12, IgG1/λ) is a fully human anti-IGF-1R antibody that binds to IGF-1R with nanomolar affinity blocking the interaction between IGF-1R and its ligands, IGF-1 and -II, and induces receptor internalization and degradation^19^. Cixutumumab is effective at inhibiting tumor growth in preclinical models and in clinical trials^20–22^. Cixutumumab cross-reacts with mouse IGF-1R and was previously shown to have good safety profile in non-human primates and humans^23,24^. The clinical development of cixutumumab has largely been abandoned due to the lack of improvement in tumor control compared with standard of care, which warrants the development of more potent immunoconjugates using this antibody. Recent data show that ADCs developed using PEG-DM1 are more potent, and can be loaded with a high drug to antibody ratio (DAR) than those developed with non-PEGylated DM1. In this study we investigate the effects of multiple PEG drugs on antibodies using IGF-1R positive cells and mouse models.

We have developed two ADCs: cixutumumab-PEG_6_-DM1-Low (3.4 DAR) and cixutumumab-PEG_6_-DM1-High (7.2 DAR). We have radiolabeled the ADCs with ^111^In via a DOTA chelator and studied the in vitro binding characteristics and in vivo behaviors using single photon emission computed tomography/computed tomography (microSPECT/CT) and ex vivo gamma counting techniques. We have also evaluated the in vivo efficacy of the ADCs in mice bearing IGF-1R positive breast cancer xenograft.

## Results

### Synthesis of drug linker and ADCs

The synthesis of drug linker DM1-MAL-PEG_6_-NHS and immunoconjugates cixutumumab-PEG_6_-DM1-Low, cixutumumab-PEG_6_-DM1-High, and controls are outlined in Supplementary Fig. 1A,B. The drug linker DM1-MAL-PEG_6_-NHS was characterized by NMR and mass spectroscopy. Data from the NMR spectra is in excellent agreement with their assigned structures (Supplementary Fig. 2). The mass spectra of these compounds showed molecular ion peaks corresponding to their molecular masses (Supplementary Fig. 3). The optimal reaction condition for antibody drug conjugation was 100 mM HEPES, pH 8.0, for 2 h at 37 °C, followed by at 4 °C for 20 h which gave the desired DAR. UV spectrophotometer was used to determine DAR and found 3.4 ± 0.3 (n = 10) for cixutumumab-PEG_6_-DM1-Low and 7.2 ± 0.1 (n = 10) for cixutumumab-PEG_6_-DM1-High. The spectra of conjugated and unconjugated IgG were sufficiently different and indicated the absorption maxima of the drug and the antibody. Cixutumumab-PEG_6_-DM1-Low and cixutumumab-PEG_6_-DM1-High showed a distinct difference in the UV absorbance at 254 and 280 nm.

### Conjugation and quality control of ADCs

Following conjugation of the antibody and ADCs with DOTA, the resulting DOTA-cixutumumab, DOTA-cixutumumab-PEG_6_-DM1-Low and DOTA-cixutumumab-PEG_6_-DM1-High (Supplementary Fig. 1) were characterized for stability, binding to recombinant IGF-1R, aggregation and molecular weight. Final conjugates resulted in clear solution with no particulate matter or milky appearance. Cixutumumab, cixutumumab-PEG_6_-DM1-Low, cixutumumab-PEG_6_-DM1-High, DOTA-cixutumumab, DOTA-cixutumumab-PEG_6_-DM1-Low, and DOTA-cixutumumab-PEG_6_-DM1-High were > 95% pure on HPLC (Supplementary Fig. 4) and bioanalyzer. The molecular weights of cixutumumab, cixutumumab-PEG_6_-DM1-Low, cixutumumab-PEG_6_-DM1-High, DOTA-cixutumumab, DOTA-cixutumumab-PEG_6_-DM1-Low, and DOTA-cixutumumab-PEG_6_-DM1-High were 146.3, 150.3, 155.7, 148.3, 152.3, and 157.7 kDa, respectively (Supplementary Fig. 5A). Bioanalyzer results indicated that there was on average three DOTA molecules per antibody.

The effect of PEG_6_-DM1 and DOTA conjugation on the binding to recombinant IGF-1R was studied using biolayer interferometry (Supplementary Fig. 5B and Table 1). The dissociation constant (K_D_) in human IGF-1R for cixutumumab, cixutumumab-PEG_6_-DM1-Low, cixutumumab-PEG_6_-DM1-High, DOTA-cixutumumab, DOTA-cixutumumab-PEG_6_-DM1-Low, and DOTA-cixutumumab-PEG_6_-DM1-High were 2.2 nM, 3.0 nM, 3.7 nM, 1.6 nM, 0.7 nM and 2.5 nM, respectively (Table 1). Cixutumumab has a high affinity to murine IGF-1R with a K_D_ of 3.5 nM (Supplementary Fig. 5B).

**Table 1:** Binding constants (K_D_) association constants (K_on_) and dissociation constants (K_diss_), by biolayer interferometry of antibody and immunoconjugates.

Immunoconjugate	K_D_ (nM)	kon (1/Ms)	kdis (1/s)
Cixutumumab	2.2	6.13E+04	1.33E−04
Cixutumumab-PEG_6_-DM1-Low	3.0	3.65E+04	1.08E−04
Cixutumumab-PEG_6_-DM1-High	3.7	3.59E+04	1.33E−04
DOTA-cixutumumab	1.6	3.41E+04	5.59E−05
DOTA-cixutumumab-PEG_6_-DM1-Low	0.7	3.07E+04	2.12E−05
DOTA-cixutumumab-PEG_6_-DM1-High	2.5	3.28E+04	8.26E−05

**Figure 1:**
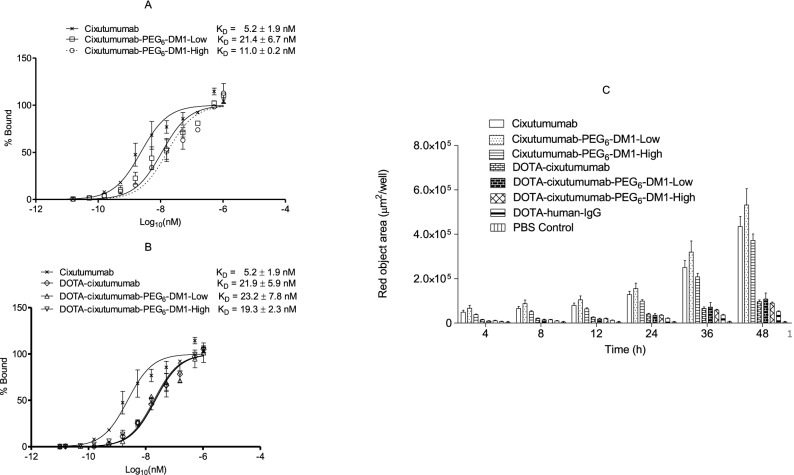
(**A**,**B**) Flow cytometry of cixutumumab conjugates in IGF-1R positive MCF-7/Her18 breast cancer. Percentage of bound was estimated from the relative fluorescence intensity and plotted against concentration. (**C**) Internalization of cixutumumab antibody conjugates in MCF-7/Her18 cells. All data shown as a mean of 6 wells ± SEM.

Saturated binding of immunoconjugates were studied in MCF-7/Her18 cells using flow cytometry. Mean fluorescence intensity (MFI) was converted into percent bound (Fig. 1A,B) and plotted against concentration to calculate EC_50_ values for MCF7/Her18 cells. Estimated EC_50_ values for cixutumumab, cixutumumab-PEG_6_-DM1-Low, cixutumumab-PEG_6_-DM1-High, DOTA-cixutumumab, DOTA-cixutumumab-PEG_6_-DM1-Low, and DOTA-cixutumumab-PEG_6_-DM1-High were 5.2 ± 1.9, 21.3 ± 6.6 nM, 11.0 ± 0.2 nM, 21.9 ± 5.9 nM, 23.2 ± 7.8 nM and 19.3 ± 2.3 nM, respectively. All the constructs were shown twofold and fourfold increase in the K_D_ values in comparison to cixutumumab. There was no significant difference in binding between cixutumumab and cixutumumab-PEG_6_-DM1-Low (5.2 ± 1.9 vs 21.4 ± 6.7; *p* > *0.05*), cixutumumab and cixutumumab-DOTA (5.2 ± 1.9 nM vs 21.9 ± 5.9 nM; *p* > 0.05) or DOTA-cixutumumab-PEG_6_-DM1-Low, and DOTA-cixutumumab-PEG_6_-DM1-High (23.2 ± 7.8 nM vs. 19.3 ± 2.3 nM; *p* > 0.05). Control antibody and control antibody conjugates did not bind MCF-7/Her18 cells (Supplementary Fig. 6).

### Internalization of immunoconjugates

A rapid time-dependent increase in red fluorescence was observed with cixutumumab immunoconjugates, but not isotype control antibody or media control, from the first time point (2 h) to end point (48 h) (Fig. 1C and Supplementary Fig. 7). The red signal was observed in the cytosolic compartment of the cells, indicative of the localization and processing of the antibody in lysosomes and endosomes which was quantified as µm^2^/well. Internalization increased from 2 to 48 h and was highest at 48 h post incubation with cixutumumab-PEG_6_-DM1-Low > cixutumumab-PEG_6_-DM1-High > cixutumumab > DOTA-cixutumumab-PEG_6_-DM1-Low > DOTA-cixutumumab > cixutumumab-PEG_6_-DM1-High > DOTA-Ctrl-IgG. The DOTA-modified conjugates were not internalized as much by MCF-7/Her18 cells.

### Cytotoxicity of ADCs

Live cell imaging was used to study the cytotoxicity of cixutumumab, cixutumumab-PEG_6_-DM1-Low, and cixutumumab-PEG_6_-DM1-High in MCF-7/Her18 breast cancer cells (Table 2). The dose response was determined using the number of viable cells after 48 h. Cixutumumab-PEG_6_-DM1-Low and cixutumumab-PEG_6_-DM1-High, were expected to be have a larger antigrowth effect than cixutumumab (Table 2). The EC_50_ of cixutumumab, cixutumumab-PEG_6_-DM1-Low and cixutumumab-PEG_6_-DM1-High were 47.1 ± 0.8 nM, 20.4 ± 0.8 nM, and 39.7 ± 0.4 nM, respectively against MCF-7/Her18 cells. The EC_50_ value of cixutumumab-PEG_6_-DM1-Low in MCF-7/Her18 was lower when compared with cixutumumab (20.4 ± 0.8 nM) and cixutumumab-PEG_6_-DM1-High (39.7 ± 0.4 nM). All these values were statistically significant from each other group (*p* < 0.05).

**Table 2:** EC_50_ values of cixutumumab, cixutumumab-PEG_6_-DM1-Low and cixutumumab-PEG_6_-DM1-High on MCF-7/Her18 breast cancer cells.

Immunoconjugates	EC_50_ (nM)^b^
Cixutumumab	47.1 ± 0.8
cixutumumab-PEG_6_-DM1-Low	20.4 ± 0.8
cixutumumab-PEG_6_-DM1-High	39.7 ± 0.4

The potency of the conjugates was estimated by plotting the increased cytotoxic red intensity as a function of cell death against concentration. Sigmoidal dose response curves (variable slope) were generated GraphPad Prism V. 5.02 (GraphPad Software Inc.). Values are the mean of triplicates of at least two independent experiments.

### Radiolabeling and characterization of radioimmunoconjugates

The radiochemical yields of ^111^In-cixutumumab, ^111^In-cixutumumab-PEG_6_-DM1-Low, and ^111^In-cixutumumab-PEG_6_-DM1-High were 79.5 ± 3.8%, 77.4 ± 2.9% and 75.5 ± 4.8%, respectively at a specific activity of 0.5 MBq/µg. A radiochemical purity of > 95% was obtained after purification of theses tracers (Supplementary Fig. 8). To investigate stability, ^111^In-DOTA-cixutumumab, ^111^In-cixutumumab-PEG_6_-DM1-Low, and ^111^In-cixutumumab-PEG_6_-DM1-High (Supplementary Fig. 9A,B) were analyzed at different time periods by HPLC following incubation at 37 ºC in PBS (Supplementary Fig. 9A) and plasma (Supplementary Fig. 9B). All tracers were > 90% stable in PBS after 72 h incubation at 37 °C. Additionally, about 10% loss of the label was observed in plasma at 48 h post incubation. The immunoreactive fraction of ^111^In-DOTA-cixutumumab, ^111^In-cixutumumab-PEG_6_-DM1-Low, and ^111^In-cixutumumab-PEG_6_-DM1-High was 77%, 75%, and 71%, respectively (Supplementary Fig. 10).

### MicroSPECT/CT and biodistribution

**Figure 2:**
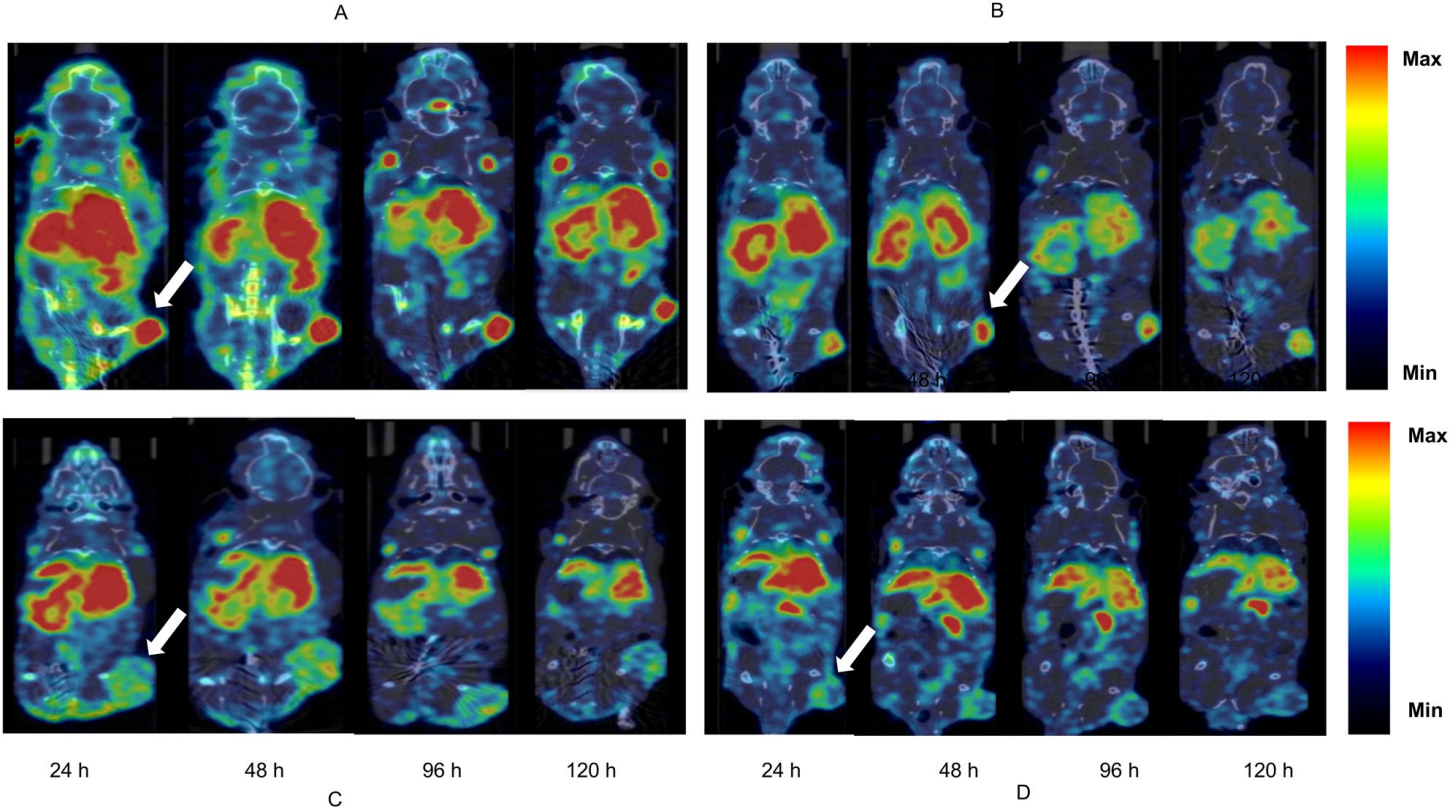
(**A**–**D**) Representative microSPECT/CT fused images of mice bearing MCF-7/Her18 xenograft at different time points after intravenous injection of (**A**) ^111^In-cixutumumab, (**B**) ^111^In-cixutumumab-PEG_6_-DM1-Low, (**C**) ^111^In-cixutumumab-PEG_6_-DM1-High and (**D**) ^111^In-human-IgG. Arrows on SPECT images indicate location of implanted tumor.

**Figure 3:**
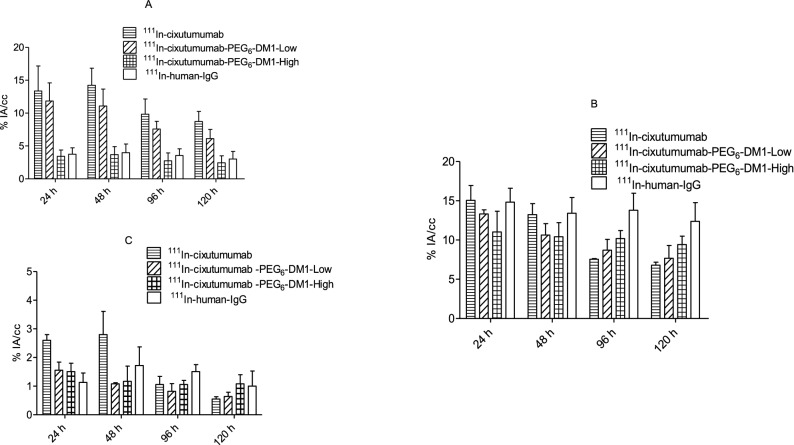
(**A**–**C**) Quantification of tracer uptake. Time-activity curves of tumor (**A**), liver (**B**), and muscle (**C**). Data are expressed as mean %IA/cc ± SD.

MicroSPECT imaging showed persistently high tumor uptake in MCF-7/Her18 xenografts (Fig. 2A–D). There were some differences in tumor uptake for ^111^In-cixutumumab, ^111^In-cixutumumab-PEG_6_-DM1-Low, and ^111^In-cixutumumab-PEG_6_-DM1-High over time. Uptake of ^111^In-cixutumumab was clearly visible as early as 24 h p.i., peaked at around 48 h p.i., and started clearing afterwards (13.4 ± 3.1, 14.4 ± 1.7, 9.8 ± 0.8, and 8.7 ± 1.3% IA/cc at 24, 48, 96, and 120 h p.i., respectively; Fig. 3B,C). Tumors were also delineated with ^111^In-cixutumumab-PEG_6_-DM1-Low (11.8 ± 2.8, 11.1 ± 2.6, 7.6 ± 1.2, and 6.1 ± 1.4% IA/cc at 24, 48, 96, and 120 h p.i., respectively; Fig. 3B). Tumor uptake of ^111^In-cixutumumab, and ^111^In-cixutumumab-PEG_6_-DM1-Low were significantly higher (*p* < 0.01) than for ^111^In-cixutumumab-PEG_6_-DM1-High at all imaging time points. The tumor uptake of ^111^In-human-IgG in MCF-7/Her18 was low (3.8 ± 1.0, 3.8 ± 1.3, 3.6 ± 1.0, and 3.0 ± 1.1% IA/cc at at 24, 48, 96, and 120 h p.i., respectively).

Liver uptake of ^111^In-cixutumumab was similar to that of ^111^In-cixutumumab-PEG_6_-DM1-Low, and ^111^In-cixutumumab-PEG_6_-DM1-High at early time points—24 and 48 h p.i. (Fig. 3B). At later time points (96 h and 120 h p.i.) the liver uptake of ^111^In-cixutumumab-PEG_6_-DM1-High was highest, followed by ^111^In-cixutumumab-PEG_6_-DM1-Low, although this trend was not statistically significant. Due to the increase in tumor uptake and clearance from the circulation, tumor-to-normal tissue contrast increased over time.

**Figure 4:**
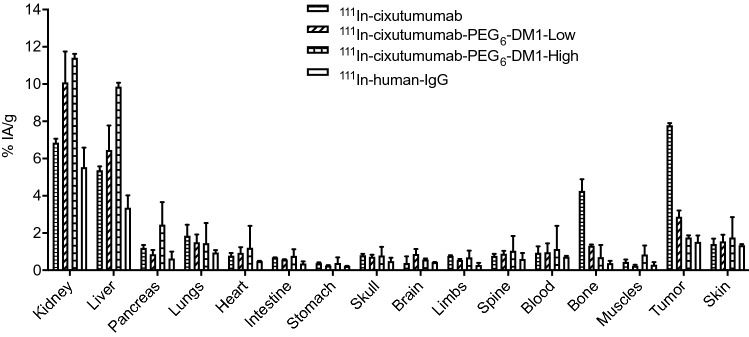
Biodistribution of ^111^In-cixutumumab, ^111^In-cixutumumab-PEG_6_-DM1-Low, ^111^In-cixutumumab-PEG_6_-DM1-High and ^111^In-Ctrl-IgG tracers in nude mice bearing MCF-7/Her18 xenografts at 144 h post injection. Data are %IA/g, expressed as mean ± SD.

All mice were euthanized after the last imaging time point (144 h p.i.) for biodistribution studies, to validate the in vivo SPECT data (Fig. 4). At 144 h p.i., ^111^In-cixutumumab was almost completely cleared from all organs except for the liver (5.4 ± 0.2%IA/g) and kidney (6.9 ± 0.2%IA/g). There was a low residual bone uptake of the ^111^In-cixutumumab-PEG_6_-DM1-High, and ^111^In-cixutumumab-PEG_6_-DM1-Low compared to ^111^In-cixutumumab with the highest uptake observed at 144 h time point. The highest absolute tumor uptake (7. 8 ± 0.1%IA/g) was found for ^111^In-cixutumumab. The absolute tumor uptake of ^111^In-cixutumumab- PEG_6_-DM1-Low (2.9 ± 0.3%IA/g) was lower than that of ^111^In-cixutumumab, and higher than ^111^In-cixutumumab- PEG_6_-DM1-High (1.8 ± 0.1%IA/g). Tumor-to-muscle ratio by biodistribution for ^111^In-cixutumumab was 17.3 ± 0.9 at 144 h p.i. (n = 3; Supplementary Fig. 11).

### Efficacy of immunoconjugates in a mouse xenograft model

**Figure 5:**
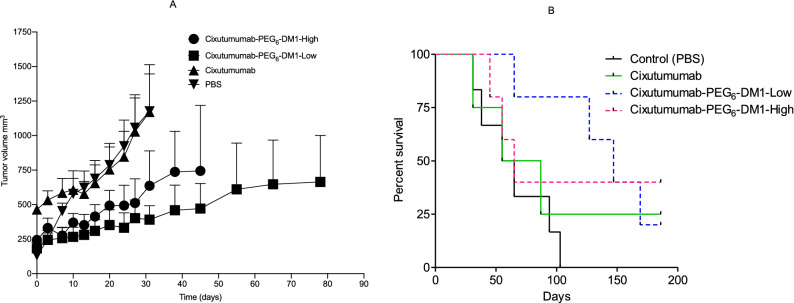
(**A**,**B**) Efficacy of cixutumumab antibody drug conjugates in IGF-1R positive mouse xenograft. (**A**) Tumor growth curves of each mouse treated with PBS, cixutumumab, cixutumumab-PEG_6_-DM1-Low, or cixutumumab-PEG_6_-DM1-High. Plots represent tumor volume (mm^3^) with time (days). The last point on the plot is when a mouse in the group reaches the 2000 mm^3^, ≥ 20% loss in body weight and/or ulcer (≥ 20% tumor volume) develops endpoint. No mouse had ≥ 20% loss in bodyweight. (**B**) Kaplan–Meier survival curves. Study endpoint occurred when xenograft volume reached 2000 mm^3^, or > 20% ulcerated. Median survival of cixutumumab-PEG_6_-DM1-Low treated mice (147 days) was significantly longer (*p* < 0.05) that cixutumumab-PEG_6_-DM1-high, cixutumumab or PBS treated mice.

We studied the in-vivo efficacy of cixutumumab, cixutumumab-PEG_6_-DM1-Low, and cixutumumab-PEG_6_-DM1-High in IGF-1R positive MCF-7/Her18 xenograft at a dose of 2.5 mg/kg (Fig. 5A). In the PBS treated group six mice had reached the study end point (2000 mm^3^) by day-103 and were sacrificed. In the cixutumumab group one mouse was alive at day 185. The one mouse showed some response to cixutumumab therapy evidenced by slowed xenograft growth. In the cixutumumab-PEG_6_-DM1-Low, and cixutumumab-PEG_6_-DM1-High groups, 2/5 mice showed response to therapy that lasted throughout the study (185 days). Tumor growth index of individual mice for the different groups is presented (Supplementary Fig. 12). No unwanted treatment-related deaths occurred in any group. Mice maintained or gained weight in the course of treatment (Supplementary Fig. 13).

Kaplan Meier survival curves showed significant differences in median survival between groups (Fig. 5B). Median survival in the PBS group was significantly (*p* < 0.05) lower than for the cixutumumab treatment groups. The median survival of the PBS group was 60 days, while that of cixutumumab group was 71 days. On the other hand, median survival of the cixutumumab-PEG_6_-DM1-Low and cixutumumab-PEG_6_-DM1-High groups 147 and 65 days, respectively.

## Discussion

The initial excitement on the use of anti-IGF-1R antibodies to treat cancers has lived up to the hype in patients^9,10^. Therefore, other approaches aimed at targeting this receptor could yield important benefits to patients. Our study is the first to describe a cixutumumab ADC. ADCs show improved efficacy compared with naked antibodies. However, cancer cells invariable develop resistance to ADCs. A major cause of resistance to ADCs is the expression of drug efflux *multidrug resistant gene* (MDR1) pumps^16–18^. Like most small molecule chemotherapeutic agents, DM1 is an MDR1 substrate, however, PEGylated DM1 (PEG-DM1) used in this study has the same potency as DM1 but is not a substrate for MDR1^18,25^. A careful balance between the DAR, therapeutic window and optimum in vivo distribution is needed for ADCs, and this may vary with different antibodies. Zhao et al*.* prepared ADCs with a DAR of 9 using PEG linkers and demonstrated that the high DAR yielded more effective ADCs in vitro that those with DAR of 3–4^18^. We developed cixutumumab-PEG_6_-DM1-Low and cixutumumab-PEG_6_-DM1-High ADCs with DARs of 3.4 and 7.2, respectively. Although physico-chemical characterization of these PEGylated-DM1 ADCs have been recently done, no studies have characterized these pegylated-DM1 ADCs in real time using imaging technologies. In the current study, we used in vitro assays, non-invasive real-time microSPECT/CT imaging and ex vivo biodistribution to characterize the behavior of PEGylated cixutumumab ADC immunoconjugates by radiolabeling the cixutumumab-PEG_6_-DM1-Low/High with ^111^In using a DOTA chelator.

To obtain a high DAR, commonly used linkers such as *N*-succinimidyl-4-(2-pyridyldithio)butanoate (SPDB) or succinimidyl-4-(*N*-maleimidomethyl)cyclohexane-1-carboxylate (SMCC) lead to the formation of aggregates that tend to abrogate the binding of the antibody^26,27^. The use of NHS-PEG_6_-DM1 resulted in ADCs with no aggregation as confirmed by HPLC and bioanalyzer. These ADCs preserved their binding to IGF-1R as confirmed by flow cytometry, and biolayer interferometry. The DOTA conjugation on cixutumumab (21.9 ± 5.9 nM) led to increase fourfold increase in K_D_ compared with the unconjugated antibody-cixutumumab (5.2 ± 1.9 nM) as observed by flow cytometry. A similar effect was observed when DOTA was conjugated to cixutumumab ADCs. (Conjugation of DOTA on ADC constructs decreased the internalization rate of all constructs (Fig. 1C). The decrease in internalization rate of the DOTA immunoconjugates may be the result of additional negative charges introduced by DOTA. The introduction of positive charges on antibodies has been shown previously to significantly improve binding and internalization rates^28^. However, this effect was expected to be less when the DOTA conjugated antibody is complexed with ^111^In (due to overall net charge), as used in imaging and biodistribution studies. At that point, factors other than the charge of the antibody play a predominant role in tissue uptake.

To be potent, ADCs developed using non-cleavable linkers/drugs must be cleaved in lysosomes to release the cytotoxin PEG_6_-DM1. Using an anti-EpCAM antibody, Kovtun et al*.* showed that there was no difference in the lysosomal processing of PEG_4_-DM1 compared to DM1 conjugated ADCs^17^. Some literature reports suggest ADCs internalize better than the naked antibodies while other reports show no differences or even the opposite^28^. This depends on the antibody–antigen complex as well as other adapter molecules. Smith et al. observed higher internalization rate using anti-melanotransferrin ADC L49 conjugated to auristatin than for the naked antibody^29^. Furthermore, Law et al*.* using anti-CD20 antibody rituximab showed that the internalization rate was dependent on drug payload with rituximab conjugated to doxorubicin (rituximab-vcDox) showing better internalization than rituximab conjugated to monomethyl auristatin E (rituximab-vcMMAE) in CD20 positive cells^30^. We showed by live cell imaging that in the case of IGF-1R internalization rate of cixutumumab-PEG_6_-DM1-Low was higher at all times compared with the unconjugated antibody (Fig. 1C).

Cixutumumab cross reacts with murine IGF-1R (Supplementary Fig. 5C) with a K_D_ of 3.5 nM. Biodistribution and imaging studies show that tumor uptake in mice bearing MCF-7/Her18 xenograft decreases with increasing number of PEG_6_-DM1 on the antibody. Tumor uptake for ^111^In-cixutumumab was slightly higher than for ^111^In-cixutumumab-PEG_6_-DM1-Low even though this was not statistically significant (*p* > 0.05). However, tumor uptake of ^111^In-cixutumumab was significantly higher (*p* < 0.0001) than ^111^In-cixutumumab-PEG_6_-DM1-High, indicating a lower binding to IGF-1R of the radioimmunoconjugate in vivo. The microSPECT imaging further confirmed the significantly lower tumor uptake of ^111^In-cixutumumab-PEG_6_-DM1-High at 24, 48, 96 and 120 h post injection.

As expected, cixutumumab ADCs were more cytotoxic than cixutumumab to MCF-7/Her18 cells. Compared with cixutumumab and cixutumumab-PEG_6_-DM1-high, cixutumumab-PEG_6_-DM1-Low had the highest internalization rate and the lowest EC_50_ (most potent in vitro). Despite having similar K_D_ values in vitro, the lower potency of cixutumumab-PEG_6_-DM1-high may be due to its slightly lower internalization rate. We studied the efficacy of the antibody and ADCs in MCF-7/Her18 xenograft. Cixutumumab-PEG_6_-DM1-Low (median survival of 147 days) prolonged the survival of tumor bearing mice compared with cixutumumab (median survival of 71 days) and cixutumumab-PEG_6_-DM1-High (median survival 65 days). Perez et al*.* showed that trastuzumab emtansine (trastuzumab-DM1) was not superior to trastuzumab plus taxanes as a first line treatment in HER2 positive breast cancer patients^31^. It remains to be seen in future preclinical studies if cixutumumab-PEG_6_-DM1-Low is inferior/superior to cixutumumab plus chemotherapeutics such as taxanes. Cixutumumab-PEG_6_-DM1-High was less effective despite having a higher drug to antibody ratio, likely due to the lower tumor uptake of the construct. Since cixutumumab cross reacts with mouse IGF-1R (Supplementary Fig. 5C) one would expect any potential toxicity issues in humans to be evident in our mouse studies. A more detailed toxicity study needs to be performed in IND enabling studies to fully determine the maximum tolerated dose of these new immunoconjugates.

## Materials and methods

### General

All experiments were performed in accordance with the University of Saskatchewan’s guidelines and regulations. Chemicals used in the conjugation, radiolabeling, and purification steps were American Chemical Society reagent grade or better. Water and buffers were rendered metal-free by passing through a column of Chelex-100 resin, 200–400 mesh (Bio-Rad Laboratories, Inc.), and were sterile-filtered through a 0.22 µm filter device. Cixutumumab was purchased from Creative Biolabs (Shirley, NY). DM1 drug was obtained from Toronto Research chemicals (Toronto, ON) and NHS-PEG_6_-Maleimide was purchased from Biochempeg (Watertown, MA). Bifunctional chelating agent 2-(4-isothiocyanatobenzyl)-1,4,7,10-tetraazacyclododecane-1,4,7,10-tetraacetic acid (*p*-SCN-Bn-DOTA) was obtained from Macrocyclics (Plano, TX). Recombinant human and mouse IGF-1R were purchased from R&D Systems (Minneapolis, MN). ^111^In was received from Nordion (Kanata, ON) as ^111^InCl_3_ in 0.1 M hydrochloric acid (optima grade, Sigma-Aldrich, St. Louise, MO). The ^1^H NMR spectra were recorded on a DPX-500 MHz Bruker FT-NMR spectrometer (St. Louis, Missouri) using CDCl_3_ as solvent. The chemical shifts were reported as parts per million (δ ppm) tetramethylsilane (TMS) as an internal standard. Mass spectra were obtained on an Agilent 6550 iFunnel QTOF mass spectrometer (Palo Alto, CA). The progress of the drug linker reaction was monitored on ready-made silica-gel plates (Merck, Etobicoke, ON) using ethyl acetate and hexane (3:1) as solvent^32^. Iodine was used as a developing agent or by spraying with the potassium permanganate reagent. Chromatographic purification was performed on a silica gel (100–200 mesh). The residues were obtained recrystallized by the addition of 30:70 hexane–chloroform. All chemicals and reagents obtained from Sigma-Aldrich (St. Louise, MO) were used without further purification^32^.

### Cell culture

The IGF-1R-positive human breast cancer cell line MCF-7/Her18 was kindly provided by Dr. Hung Mien-Chie (M.D. Anderson Cancer Center, Houston, TX, USA)^33^. MCF-7/Her18 is MCF-7 cell line that has been transfected to express HER2^34^. The role of IGF-1R in resistance to targeted HER2 therapies has been extensively studied preclinically using MCF-7/HER18 cell line^6^. MCF-7/HER18 is isogenic with MCF-7 except for the expression of HER2, and xenografts established using these cells have similar growth rates. MCF-7/HER18 cell line has medium IGFR-1R expression with ~ 100,000 receptors/cell^34–36^. MCF-7/Her18 cells were maintained in Dulbecco’s minimal essential medium containing high glucose levels and 10% fetal bovine serum (FBS). Cells were grown in a humidified atmosphere with 5% CO_2_ at 37 °C.

### Quality control of immunoconjugates

The purity of the antibody conjugates was determined by size exclusion HPLC (Waters 2796 Bioseparations Module, Waters 2487 Dual λ Absorbance Detector, XBridge^®^ BEH 200A SEC 3.5 µm 7.8 × 300 mm column, Waters Corporation)^32^. The UV-Detector was set at 220 and 280 nm and the solvent system was phosphate buffered saline (PBS) at a flow rate of 0.6 mL/min.

#### Bioanlzyer

The analysis of molecular weight and purity of the immunoconjugates was performed on an Agilent 2100 Bioanalyzer using Agilent High Sensitivity Protein 250 Kit (cat # 5067-1575) following the manufacturer’s protocol. The size and relative peak area were calculated using Agilent 2100 Expert software^32^.

#### Biolayer interferometry

Binding kinetics between the antibodies and IGF-1R were measured using biolayer interferometry (BLI) with ForteBio Octet RED384 (PALL Corporation, CA). Antibodies were immobilized on Anti-human IgG Fc Capture sensors (18-5060, Forte Bio) according to manufactures instructions. The equilibrium dissociation constant (K_D_) was obtained using a 1 to 1 binding model with local fitting. Data analysis and curve fitting was performed using data analysis software 7.1.0.33 (Forte Bio)^33^.

#### Flow cytometry

MCF-7/Her18 cells were treated with antibodies at twelve concentrations 1000–0.015 nM, and incubated for 30 min at room temperature followed by 15 min on ice. Cells were washed with PBS + 2% FBS and incubated with secondary antibody FITC labeled Goat F(ab’)_2_ fragment anti-human IgG (H + L) (1:50 ratio) (IM0839, Beckman Coulter) for 30 min at 4 °C, then washed again. Flow data was acquired using a Beckman Coulter Gallios flow cytometer (Beckman Coulter) and analyzed by FlowJoV10. 6 the EC_50_ was determined using GraphPad Prism 6.

### Internalization of immunoconjugates

Internalization was studied using a previously reported SOP^33^. MCF-7/Her18 cells (10,000 per well) were plated in flat-bottom 96 well plates and incubated 12 h prior to the assay using IncuCyte S3 live cell imaging system (Essen BioScience, Ann Arbor, MI). Briefly, cixutumumab immunoconjugates or control human-IgG isotype at known concentration (4 µg/mL) mixed with IncuCyte FabFluor reagent (Essen BioScience, Ann Arbor, MI) at a molar ratio of 1 to 3 in complete growth media. Reactions were performed at twice the required final concentration in a round bottom 96 well plate depending on the volumes required and incubated for 15 min at 37 °C. Images were captured every 2 h using a 10× objective lens by a phase contrast and fluorescence channel and quantified as described previously^32^.

### Radiolabeling and immunoreactivity

Radiolabeling of DOTA conjugates with ^111^InCl_3_ was performed as reported earlier^37^. The reaction was monitored using iTLC using 100 mM sodium citrate buffer (pH 5.0) as mobile phase. The iTLC strips were analyzed using ScanRam (LabLogic, Brandon, FL). The ^111^In-labeled conjugates were purified using Amicon Ultra-4 centrifugal filters (10 K, EMD Millipore, Burlington, MA) with PBS, and purity was determined using size exclusion radio-HPLC and iTLC. A radiochemical purity (RCP) > 95% was considered good for in vitro and in vivo studies. The immunoreactive fraction of the ^111^In-labeled immunoconjugates was determined using MCF-7/Her18 cells as previously described^38^.

### In vitro cytotoxicity of ADCs

The in vitro cytotoxicity (EC_50_ values) of cixutumumab immunoconjugates was determined using IncuCyte S3 live cell imager^33^. Briefly, 3000–5000 (MCF-7/Her18) cells were seeded 24 h prior to treatment in a 96 well plates. Media was removed and cells were washed with PBS. Cells were then incubated with IncuCyte^®^ Cytotox Red reagent diluted in complete media (1× , Essen Bioscience Cat #4632) for 3 h before treatment. Then, cells were treated with different concentrations (0.2–500 nM) of the antibodies and ADCs at 37 °C for 30 min prior to imaging. Live cell imaging was performed as described above and the EC_50_ values for individual compounds were calculated using GraphPad Prism 5.

### Tumor xenograft, microSPECT/CT imaging and biodistribution

Animal studies were approved by the University of Saskatchewan Animal Care and Use Committee as per protocol # 20170084. Female athymic CD-1 nude mice were purchased from Charles River Laboratory (Sherbrooke, QC). The mice were housed under standard conditions in approved facilities with 12 h light/dark cycles and provided ad libitum throughout the duration of the studies. For inoculation, MCF-7/Her18 cells (5 × 10^7^ cells/mL) were resuspended in a 1:1 mixture of PBS: Matrigel (BD Biosciences, ON) and 0.2 mL suspension was injected in the right flank. The radiolabeled antibodies were passed through 0.22 µm Ultra free MC filter and 10–12 MBq (specific activity, 0.5 MBq/µg) was injected via tail a vein in tumor bearing mice.

MicroSPECT images were acquired using Vector4CT scanner (MILabs, Utrecht) at 24, 48, 96, and 120 h post injection using a similar lab protocols^39^. The mice were anesthetized using a mixture of isoflurane/oxygen (5% of isoflurane in oxygen) and whole-body SPECT/CT images were obtained while anesthesia was maintained at 2%. Body temperature, heart rate, and breathing frequency were monitored continuously using physiosuite (Kent Scientific). All the scans were acquired in a list-mode data format with an ultra-high sensitivity (XUHS-2.0 mm) mouse pinhole collimator with 75 pinholes^40,41^. Corresponding CT scans were acquired with a tube setting of 50 kV and 480 μA, single frames of full scan angle were acquired in 480 steps and shoot rotation mode.

SPECT image reconstruction was carried out following a similar protocol as reported recently^42,43^. For quantification, SPECT images were analyzed using PMOD 3.8 biomedical image analyzing software (PMOD, Switzerland). Region-of-interest (ROI) was manually drawn to encompass the radioactivity uptake in the organ of interest. Tracer uptake was expressed as % IA/mL of tissue volume (% IA/cc). The result was reported as mean ± standard deviation within each study group.

At the end of imaging (144 h), mice (n = 4 per group) were sacrificed and tissues were harvested for gamma counting, and radioactivity in the organs was expressed as % injected activity per gram (%IA/g).

### Efficacy of ADCs in mouse xenograft

When xenografts average size measured 250–350 mm^3^ in volume, mice were randomized into ≥ 4 mice per group and each mouse was injected intravenously via a tail vein with normal saline or three doses (~ 2.5 mg/kg) of cixutumumab, cixutumumab-PEG_6_-DM1-Low or cixutumumab-PEG_6_-DM1-High on day 0, 4, and 8. Tumor growth was monitored using a digital caliper and growth curves were established using the tumor volumes. The study was terminated when xenograft reached a volume ≥ 2000 mm^3^ and this was used to estimate the Kaplan Meier survival curves in the different groups. Individual body weight of mouse (every other day) was recorded during the quarantine and experimental period. At the dose selected, gross examination of organs and body weights of treated mice did not show dose limiting toxicity.

### Statistical analysis

All data was expressed as the mean ± SD or SEM of at least 3 independent experiments. Statistical comparisons between the experimental groups were performed 1-way ANOVA with Bonferoni multiple comparison post hoc test (multiple-group comparison). Graphs were prepared and *P* values calculated by using GraphPad Prism (version 5; GraphPad, La Jolla, CA). *P* values of less than 0.5 considered significant.

## Conclusion

Using PEG_6_-DM1, we developed cixutumumab ADCs with low (cixutumumab-PEG_6_-DM1-Low: DAR 3.4) and high (cixutumumab-PEG_6_-DM1-High: DAR 7.2) DARs. We used microSPECT imaging and ex vivo biodistribution to understand the tumor uptake and distribution of antibody conjugates. Unlike the cixutumumab-PEG_6_-DM1-High, cixutumumab-PEG_6_-DM1-Low showed similar uptake as the unconjugated cixutumumab. This good tumor uptake and high antibody internalization rate makes cixutumumab-PEG_6_-DM1-Low more effective and warrants further investigation.

## Supplementary information

Supplementary Information.
