# Supplementary material for: Development and preclinical evaluation of cixutumumab drug conjugates in a model of insulin growth factor receptor I (IGF-1R) positive cancer
Source: Sci Rep. 2020 Oct 29;10:18549. doi: 10.1038/s41598-020-75279-z (PMC7596529; doi:10.1038/s41598-020-75279-z)
Supplement: Supplementary file 1 — Supplementary Information. [file 41598_2020_75279_MOESM1_ESM.docx]

**Development and preclinical evaluation of cixutumumab drug conjugates in a model of insulin growth factor receptor I (IGF-1R) positive cancer**

Viswas Raja Solomon^1^, Elahe Alizadeh^1^, Wendy Bernhard^2^, Amal Makhlouf ^1,3^, Siddesh V. Hartimath^1^, Wayne Hill^2^, Ayman El-Sayed^2^, Kris Barreto^2^, Clarence Ronald Geyer^2^, Humphrey Fonge^1,4*^

^1^Department of Medical Imaging, University of Saskatchewan, College of Medicine, Saskatoon SK, Canada

^3^Department of Pathology and Laboratory Medicine, University of Saskatchewan, College of Medicine, Saskatoon SK, Canada

^3^Department of Pharmaceutics and Industrial Pharmacy, Faculty of Pharmacy, Cairo University, Kasr El-Aini, 12411, Cairo, Egypt

^4^Department of Medical Imaging, Royal University Hospital Saskatoon, Saskatoon SK, Canada

***Corresponding author**

Humphrey Fonge, PhD

103 Hospital Dr.

Department of Medical Imaging

RUH Saskatoon, Saskatoon SK, S7N 0W8

Canada

Email: humphrey.fonge@usask.ca

Tel: 306-655-3353

Fax: 306-655-1637

**Supplementary information**

**Synthesis of Antibody Drug Conjugates**

Synthesis of the drug linker MAL-DM1-PEG_6_-NHS was carried out as per reported protocol[^1^](#_ENREF_1). Briefly, a solution of *N*^2’^ deacetyl-*N*^2’^-(3-mercapto-1-oxopropyl)maytansine (DM1, 28.1 mg, 0.0381 mmol) was prepared in 0.5 mL of THF. MAL-PEG_6_-NHS, (39.1 mg, 0.0762 mmol) in 1.5 mL PBS (50 mM, pH 6) was then added to the drug solution. The reaction was allowed to proceed for 1 h with stirring at room temperature. TLC was used to (ethyl acetate and hexane 3:1) to monitor the reaction. The crude reaction mixture was purified using silica gel column chromatography, eluting with 6% ethanol in methylene chloride. The solvent was removed under vacuum to give 19.2 mg (40 % yield) of the desired product. ^1^H NMR (500 MHz, CDCl_3_) δ 0.79 (3H, s), 1.19-1.27 (3H, m), 1.29 (3H, s), 1.31 (2H, d, *J* = 2.4 Hz), 1.45 (1H, m), 1.56 (1H, d, *J* = 13.6 Hz), 1.63 (3H, s), 2.17 (1H, dd, *J* = 11.6 and 2.6 Hz), 2.35 (1H, dd, J = 14.8 and 3.8 Hz), 2.44 (2H, m), 2.57-2.64 (2H, m), 2.79-2.90 (14H, m), 2.92-3.14 (5H, m), 3.19 (3H, s), 3.35 (3H, d, *J* = 1.6 Hz), 3.4 (2H, m), 3.48 (1H, d, *J* = 8.8), 3.52 (2H, m), 3.65 (13H, m), 3.71 (2H, m), 3.77 (1H, m), 3.83 (2H, t), 3.98 (3H, s), 4.27 (1H, t), 4.77 (1H, d, *J* = 12 Hz), 5.354 (1H, m), 5.64 (1H, m), 6.24 (1H, s), 6.36 (1H, broad s), 6.42 (1H, dd, *J* = 11.2 and 4 Hz), 6.63 (1H, s), 6.66 (1H, dd, *J* = 6.4 and 4.6 Hz), and 6.82 (1H, dd, *J* = 4 and 1.6 Hz). HRMS: found, 1339.5446 (M+H); calcd, 1339.8776

Cixutumumab with low (cixutumumab-PEG_6_-DM1-Low) and high (cixutumumab-PEG_6_-DM1-High) drug to antibody ratios (DAR) were prepared. A 20 mg/mL solution of the drug linker MAL-DM1-PEG_6_-NHS was prepared in DMSO. The antibody buffer was then exchanged to 100 mM 4-(2-hydroxyethyl)-1-piperazine ethane sulfonic acid (HEPES, pH 8.0) by 3 complete rounds of concentration and subsequent dilution using a size exclusion centrifugal column (Amicon Ultra-10K, Burlington MA). For the cixutumumab-PEG_6_-DM1-Low immunoconjugate, a solution of antibody (5 mg/mL) was treated with 6 to 8 equivalent of MAL-DM1-PEG_6_-NHS in HEPES buffer (0.1 M, pH 8.0). For cixutumumab-PEG_6_-DM1-High, a 14 to 16 equivalent of MAL-DM1-PEG_6_-NHS was used. The reaction mixtures were incubated at 37 ºC on a shaker at 600 RPM for 2 h and then 20 h at 4 ºC. Unreacted drug linker was removed by centrifugal filter column equilibrated in 150 mM PBS buffer containing 100 mM NaCl at pH 7.0 using an Amicron Ultra-10K molecular filtration device. The number of molecules of PEG_6_-maytansine incorporated per antibody molecule was assessed by measuring the absorbance at A_254_ and A_280_ for antibody-drug conjugates and antibody, respectively [^2^](#_ENREF_2).

Supplementary Fig. 1A. Conjugation of cixutumumab-PEG_6_-DM1 drug conjugates with *p*-SCN-DOTA and radiolabeling with ^111^Indium.

+

i)

ii)

Mal-PEG_6_-NHS

DM1

DM1-PEG_6_-NHS

Cixutumumab-PEG_6_-DM1

Supplementary Fig. 1B: Synthesis of cixutumumab drug conjugation. Maytansine (DM1) was reacted with bifunctional linker NHS-PEG_6_-Mal (2) in 50 mM PBS/THF for 6 h at room temperature to generate the NHS-PEG_6_-DM1. NHS-PEG_6_-DM1 was analyzed by mass spectrometry and NMR. Different fold excess of NHS-PEG_6_-DM1 was then reacted with cixutumumab in 0.1 M HEPES pH 8.5 at room temperature for 3 h followed by 4 ºC for 20 h to yield cixutumumab-PEG_6_-DM1-Low (3 – 4 drugs per antibody) or cixutumumab-PEG_6_-DM1-High (7 – 8 drugs per antibody).

Supplementary Fig. 2 ^1^H NMR spectra of DM1-MAL-PEG_6_-NHS

Supplementary Fig. 3 MASS spectra of DM1-MAL-PEG_6_-NHS

**Conjugation of Antibody Drug Conjugates with *p*-SCN-Bn-DOTA**

A 5 mg/mL solution of cixutumumab, cixutumumab-PEG_6_-DM1-Low or cixutumumab-PEG_6_-DM1-High in PBS was buffer exchanged to 0.1 M NaHCO_3_ (pH 9) using a centrifugal (Amicon Ultra-10K, Burlington, MA) molecular filtration device and concentrated to 10 mg/mL. A 16-fold molar excess of *p*-SCN-Bn-DOTA (DOTA) in DMSO was added dropwise to the antibody solution (final volume of DMSO was kept below 5%). The reaction mixture was incubated at room temperature on a shaker at 600 RPM for 2 h. The excess of unreacted chelator was removed by centrifugation using an Amicon Ultra-10K molecular filtration device. The resulting solution was filtered using 0.22 μm Millipore filters and stored at -80 °C for labeling with ^111^In.

Supplementary Fig. 4. Representative size exclusion (SEC) HPLC profile of immunoconjugates

Supplementary Fig. 5A. Bioanalyzer electropherograms of the ladder, cixutumumab, cixutumumab-PEG_6_-DM1-Low, cixutumumab-PEG_6_-DM1-High, DOTA-cixutumumab, DOTA-cixutumumab-PEG_6_-DM1-Low, and DOTA-cixutumumab-PEG_6_-DM1-High.

Supplementary Fig. 5B. Binding of antibodies to IGF-1R using biolayer interferometry at different concentrations (55, 166 and 500 nM).


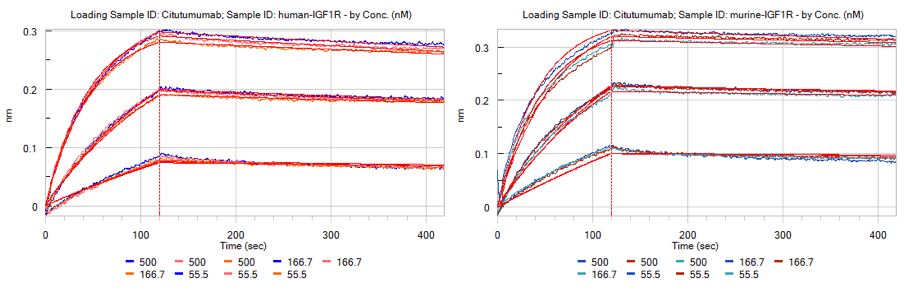


Supplementary Fig. 5C. Binding of cixutumumab to murine IGF-1R using biolayer interferometry. at different concentrations (500 – 55.5 nM).


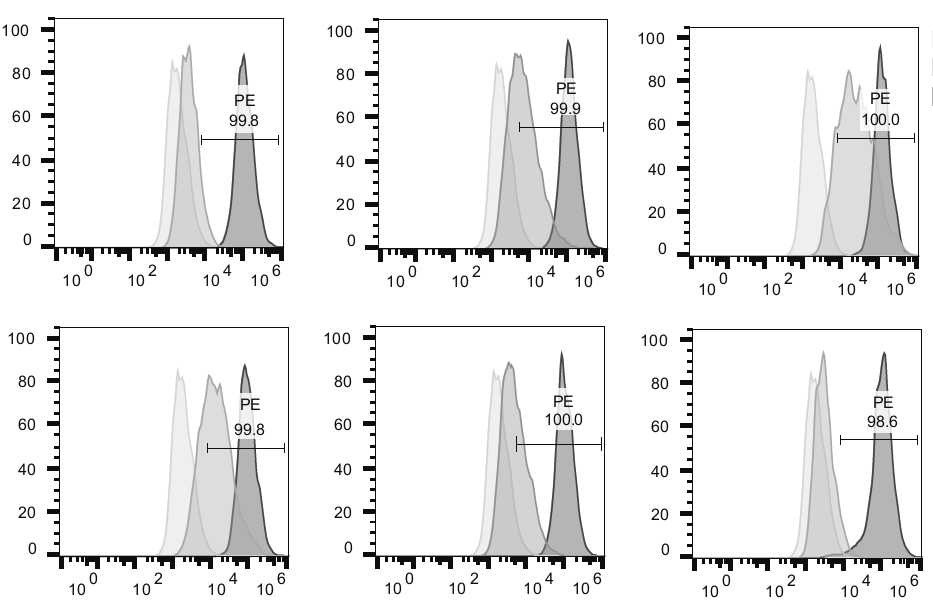


A

B

C


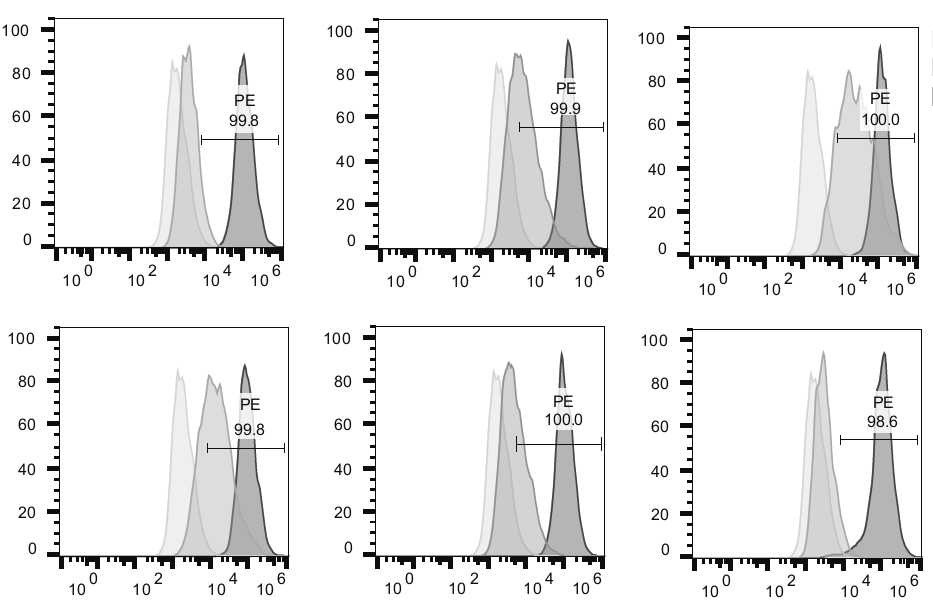


D

E

F

Fluorescence intensity

Cell Count

Cell Count

Fluorescence intensity

Supplementary Fig. 6. *In vitro* binding of immunoconjugates in IGF-1R positive MCF-7/Her18 breast cancer cells by flow cytometry. Histograms showing the binding of cixutumumab antibody and human-IgG antibody constructs at 100 nM. Light gray: unstained MCF-7/Her18 cells. Medium gray: Cells incubated with 100 nM human-IgG antibody constructs. Dark gray: Cells incubated with 100 nM cixutumumab antibody constructs. (A): Control human-IgG and cixutumumab; (B): Control human-IgG-PEG_6_-DM1-Low and cixutumumab-PEG_6_-DM1-Low; (C): Control human-IgG-PEG_6_-DM1-High and cixutumumab-PEG_6_-DM1-High; (D): DOTA-human-IgG and DOTA-cixutumumab; (E): DOTA-human-IgG-PEG_6_-DM1-Low and DOTA-cixutumumab-PEG_6_-DM1-Low; and (F): DOTA-human-IgG-PEG_6_-DM1-High and DOTA-cixutumumab-PEG_6_-DM1-High.

Supplementary Fig. 7. Internalization of IncuCyte® FabFluor labeled cixutumumab conjugates in MCF-7/Her18 cells. MCF-7/Her18 cells were treated with either IncuCyte® FabFluor labeled with cixutumumab conjugates or IgG1 isotype control (4 μg/mL), HD phase and red fluorescence images (10x) were captured every 2 h over 48 h. Images of cells treated with FabFluor-cixutumumab constructs display red, cytosolic fluorescence after 48 h.

**Stability of Radioimmunoconjugates**

The stability of the radioimmunoconjugates was evaluated *in vitro* in saline and human plasma. 50 µL of radiolabeled compound was added to 1 mL of human plasma or saline to make a final concentration of 20 MBq/mL, followed by incubation at 37 °C for up to 5 days (n = 3). Aliquots were taken at different time points and analyzed for radiochemical purity using iTLC.


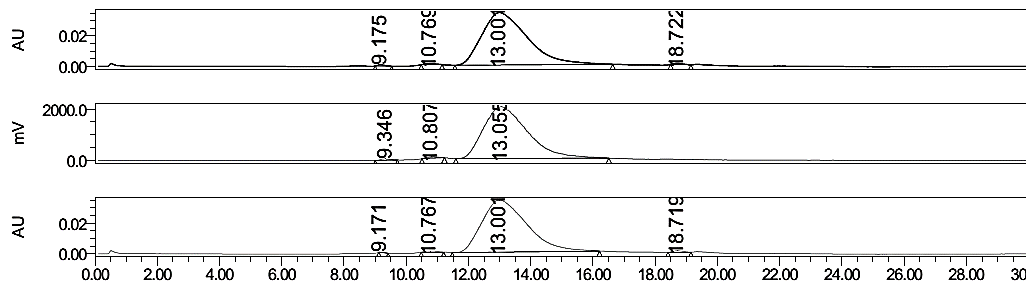

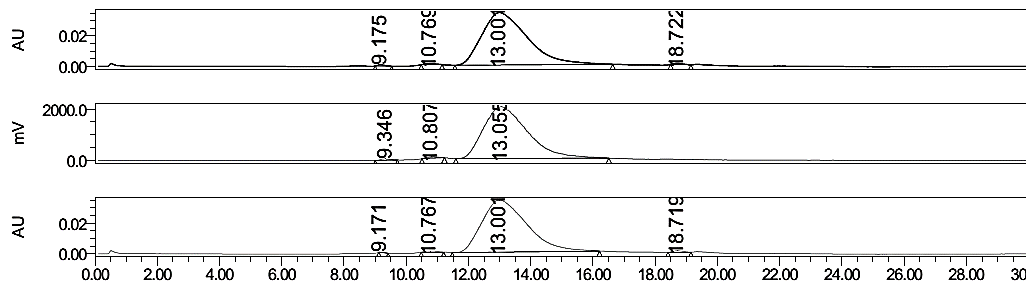


UV @280 nm

Radiometric

Supplementary Fig. 8. A representative size exclusion (SEC) HPLC profile of ^111^In-cixutumumab

Supplementary Fig. 9. *In vitro* stability of ^111^In-cixutumumab, ^111^In-cixutumumab-PEG_6_-DM1 and ^111^In-cixutumumab-PEG_6_-DM1-High in PBS (a) and human plasma (b) at 37 °C for different time points.

Supplementary Fig. 10. Immunoreactive fraction determination of ^111^In-cixutumumab, ^111^In-cixutumumab-PEG_6_-DM1-Low, and ^111^In-cixutumumab-PEG_6_-DM1-High. Binding plot of the ratio of specifically bound ^111^In-cixutumumab (a), ^111^In-cixutumumab-PEG_6_-DM1-Low (c) and ^111^In-cixutumumab-PEG_6_-DM1-High (e) to the total applied radioactivity (B/T) as a function of cell concentration (X-axis). The cell concentration is expressed as cells (million/mL). B) Lindmo plot showing total/bound activity of ^111^In-cixutumumab (b), ^111^In-cixutumumab-PEG_6_-DM1-Low (d), and ^111^In-cixutumumab-PEG_6_-DM1-High (f) as a function of inverse of cell concentration.

|  |  |
| --- | --- |

Supplementary Fig. 11. Tumor to muscle (a) and liver (b) ratios obtained from microSPECT/CT images

Supplementary Fig. 12.

Supplementary Fig. 13. Mouse body weight

**References**

1. Zhao RY, Wilhelm SD, Audette C, et al. Synthesis and evaluation of hydrophilic linkers for antibody-maytansinoid conjugates. J Med Chem. 2011;54: 3606-3623.

2. Chen Y. Drug-to-antibody ratio (DAR) by UV/Vis spectroscopy. Methods Mol Biol. 2013;1045: 267-273.
